# Protective Effects of Huang-Lian-Jie-Du-Tang against Polymicrobial Sepsis Induced by Cecal Ligation and Puncture in Rats

**DOI:** 10.1155/2013/909624

**Published:** 2013-12-04

**Authors:** Yufen Wei, Lei Shan, Liming Qiao, Runhui Liu, Zhenlin Hu, Weidong Zhang

**Affiliations:** ^1^School of Pharmacy, Second Military Medical University, 325 Guohe Road, Shanghai 200433, China; ^2^Changhai Hospital, Second Military Medical University, 174 Changhai Road, Shanghai 200433, China

## Abstract

Huang-Lian-Jie-Du-Tang (HLJDT) is a traditional formula that has long been used for treatment of inflammatory diseases in Traditional Chinese Medicine. In this study, we examined its protective effect against sepsis in an experimental septic model induced by cecal ligation and puncture (CLP) in rats. The results demonstrated that prophylactic administration of HLJDT protected rats from CLP-induced lethality and ameliorated CLP-induced liver and lung injury. HLJDT treatment suppressed the production of proinflammatory cytokines, including TNF-*α*, IL-1, IL-6, and IL-17A, indicating HLJDT could limit excessive inflammatory responses in septic condition. In addition, HLJDT facilitated bacterial clearance by increasing phagocytic activities of peritoneal macrophages. Furthermore, HLJDT treatment reversed CLP-induced suppression of IFN-*γ* expression and blocked CLP-induced increase in IL-4 expression in spleens of rats at 24 h after CLP, indicating that HLJDT could reverse the shift from Th1 to Th2 response and promote Th1/Th2 balance toward Th1 predominance in septic rats. Moreover, HLJDT also inhibited the expression of IL-17A and ROR-*γ*t in spleens of septic rats, indicating HLJDT is able to inhibit Th17 activation in septic condition. In conclusion, the present study demonstrated the protective effects of HLJDT against sepsis and highlighted the potential of HLJDT as a medication for septic patients.

## 1. Introduction

Sepsis is the systemic response of the host towards invading microorganisms and their toxins [1, 2]. Despite rapid progress in medical care over the past decades, sepsis remains a leading cause of mortality in Intensive Care Units [1, 3]. The early hallmark sign of sepsis is a whole-body inflammatory state called systemic inflammatory response syndrome (SIRS). This early phase of excessive systemic inflammation can compromise the function of distinct organ systems, leading to multiple organ dysfunction syndrome (MODS) [1, 4]. SIRS is predominantly mediated by cytokines. It is now well established that bacterial infection leads to the activation of the cytokine network [5]. In patients with sepsis, TNF-*α* is the first proinflammatory cytokine that is released, followed by others including IL-1, IL-6, and IL-8 [6, 7]. TNF-*α* and IL-1 are the most important proinflammatory cytokines, they act synergistically in activating target cells and inducing the production of more inflammatory mediators and are largely responsible for the clinical manifestations of sepsis [6, 8]. More recently, IL-17A (the first described member of the IL-17 family) was identified as a novel mediator of sepsis. As an important proinflammatory cytokine, IL-17A mediates neutrophil stimulation and T lymphocyte mobilization in sepsis [9, 10]. In addition to activating a proinflammatory cytokine cascade, inflammatory stimuli activate the production of anti-inflammatory cytokines such as IL-10, IL-4, IL-13, and TGF-*β* [11, 12]. These cytokines suppress the expression of IL-1*β* and TNF-*α*, promote a switch from Th1 to Th2 activation, and inhibit antigen presentation by monocytes as well as T and B lymphocyte function. In sepsis, the initial excessive inflammatory response is progressively counterbalanced by the negative feedback of anti-inflammatory process, which may adversely affect immune functions leading to inability to clear the infection and predisposition to secondary nosocomial infections thereby having a deleterious effect on patient outcome [13, 14].

On the assumption that endotoxin, proinflammatory cytokines, and other inflammatory mediators were responsible for the manifestations of sepsis, strategies were developed for blocking these inflammatory mediators [15, 16]. However, attempts at attenuating the inflammatory response by the neutralization of individual mediators have failed [17, 18]. Clinical trials have demonstrated that the anti-inflammatory treatments with agents such as antiendotoxin antibodies [19, 20], monoclonal antibodies against TNF [21, 22], soluble TNF receptors [23, 24], IL-1 receptor antagonist [18, 25], PAF antagonists [26, 27], or a nonselective NOS inhibitor [28] have failed to improve the outcome of patients with severe sepsis. Nevertheless, since sepsis is characterized by the release of a great variety of inflammatory mediators into the circulation, it still holds promise that combinatory therapy containing a “cocktail” of agents which modulates multiple pathogenic pathways will prove to be beneficial in the treatment of patients with sepsis [29–31]. Furthermore, the host response to sepsis involves many subsequent and concurrent processes that involve both exaggerated inflammation and immune suppression. Future therapy should emphasize both limiting systemic inflammatory response and improving host immunity against infections.

Traditional Chinese Medicine (TCM) has advocated combinatory therapeutic strategies over thousands of years. Formulae containing a combination of different kinds of herbs are often used in TCM to increase efficacy and to lower toxicity. Huang-Lian-Jie-Du-Tang (HLJDT) is a traditional formula consisting of four herbs: Rhizoma Coptidis, Radix scutellariae, Cortex Phellodendri, and Fructus Gardeniae. It has wide applications in the clinical practice of TCM. Especially, HLJDT is recognized in China as an effective anti-inflammatory agent and has been widely used in the treatment of various inflammatory diseases such as gastritis, dermatitis, and aphthous stomatitis [32]. Animal studies demonstrated that treatment with HLJDT could provide therapeutic efficacy in various inflammatory models, such as dextran sulfate sodium-induced colitis in mice [33], collagen-induced arthritis in rats [34, 35], and carrageenan-induced paw edema in mice [36]. In animal experiments, HLJDT suppressed the secretion of proinflammatory cytokines including TNF-*α*, IL-1, and IL-6 under inflammatory conditions [33]. The secretion of these cytokines was also reported to be suppressed by HLJDT *in vitro* [37]. In the present study, we examined whether HLJDT is of a potential therapeutic value for sepsis and investigated its anti-inflammatory and immune modulating roles in septic condition.

## 2. Materials and Methods

### 2.1. Medical Herbs

Rhizoma Coptidis (rhizoma of *Coptis chinensis *Franch), Radix Scutellariae (radix of *Scutellaria baicalensis *Georgi), Cortex Phellodendri (cortex of *Phellodendron chinense *Schneid), and Fructus Gardeniae (fructus of *Gardeniajasminoides* Ellis) were purchased from Bozhou (Anhui province, China) and were authenticated by Professor HanMing Zhang (Second Military Medical University, Shanghai, China). The voucher specimens were stored in Department of Natural Medicinal Chemistry, School of Pharmacy, Second Military Medical University.

### 2.2. Preparation of HLJDT

HLJDT extract was prepared as previously described [38]. In brief, Rhizoma Coptidis, Radix Scutellariae, Cortex Phellodendri, and Fructus Gardeniae were mixed in a ratio of 3 : 2 : 2 : 3. The mixture was decocted twice with boiling water (1 : 10, v/v) for 1 h, and the extracted solution was filtered through 5 layer gauzes. The filtrations were combined and freeze-dried. The lyophilized powder was stored at 4°C and dissolved in sterile distilled water just before administration. The yield of HLJDT extract was 20% (w/w) of the dried herbs. The quality of HLJDT extract is monitored by chromatographic fingerprinting as described previously [38].

### 2.3. Animals

Male Sprague-Dawley (SD) rats (3 months old, 250–300 g) were purchased from Slacom Experimental Animal Company (Shanghai, China). All animals were acclimatized under controlled temperature (20 ± 2°C), humidity (60 ± 5%) and 12 h light/12 h dark cycle for 1 week before the experiment.

### 2.4. CLP Model of Sepsis

All animal experiments were approved by the Administrative Committee of Experimental Animal Care and Use of Second Military Medical University and conformed to the National Institute of Health guidelines on the ethical use of animals. The CLP procedure was performed as described previously [39, 40]. Briefly, rats were anesthetized with an intramuscular injection of ketamine (75 mg/kg, Yuhan Corporation, Seoul, Republic of Korea) and xylazine (20 mg/kg, Bayer, Germany), a midline laparotomy was made using minimal dissection, and the cecum was ligated just below the ileocecal valve with 2-0 silk, so that intestinal continuity was maintained. The antimesenteric surface of the cecum was perforated with an 18-gauge needle at two locations 1 cm apart and the cecum was gently compressed until fecal matter was extruded. The bowel was then returned to the abdomen and the incision was closed. At the end of the operation, all rats were resuscitated with saline, 3 mL/100 g, body weight, given subcutaneously. The sham-operated groups were given a laparotomy, and the cecum was manipulated but not ligated or perforated. All animals were returned to their cages with free access to food and water.

### 2.5. Drug Administration

Rats were randomly divided into the following groups—sham group: rats underwent the sham operation and received vehicle (sterile distilled water); CLP group: rats were subjected to CLP and received vehicle; CLP + HLJDT group: rats received oral administration of HLJDT (120 or 270 mg/kg) 2 h prior to CLP.

### 2.6. Histological Analysis

The livers and lungs of the septic and sham-operated rats were harvested at 24 h after CLP. The tissue samples were fixed in 10% formalin solution, embedded in paraffin, and sectioned. The tissue sections were then stained with the hematoxylin and eosin reagent according to standard protocols and observed under light microscopy.

### 2.7. Measurement of the Plasma Levels of ALT, AST, and Cytokines

Blood samples were collected via the abdominal artery from the septic or sham-operated rats at 4 h, 12 h, and 24 h after surgery. Eighteen rats in each group were used for blood sampling, and 6 rats were used at each time point. To collect the blood samples, rats were anesthetized with an intramuscular injection of ketamine (75 mg/kg, Yuhan Corporation, Seoul, Republic of Korea) and xylazine (20 mg/kg, Bayer, Germany). A midline laparotomy was made and the abdominal artery was exposed by shifting the intestines over to the left. The blood was collected via the abdominal artery using a 23–25-gauge needle and a 5 mL syringe by inserting the needle into the artery and drawing blood slowly. Plasma was obtained after centrifugation of the samples (300 g for 5 min). ALT and AST levels were measured using commercialized kits purchased from Nanjing Jiancheng Bioengineering Institute (Nanjing, China) according to manufacturer's instructions. Levels of TNF-*α*, IL-1, IL-6, IL-17A, and IL-10 were measured using ELISA kits purchased from eBioscience (San Diego, USA) according to manufacturer's instructions.

### 2.8. Analysis of Cytokine Gene Expression by Quantitative RT-PCR (qRT-PCT)

Rats were sacrificed 24 h after CLP. The liver, lung, kidney, and spleen were harvested for RNA isolation. Total RNA was extracted with TRIzol reagent (Invitrogen, Carlsbad, CA, USA) according to manufacturer's instructions. For each sample, 500 ng of total RNA was reversely transcribed using PrimeScript RT reagent Kit (Takara, Dalian, China). PCR amplification was performed on a StepOnePlusTM real time PCR system (Applied Biosystems, USA) using the SYBR Premix Ex TaqTM PCR Kit (Takara, Dalian, China). The primers used were designed using Primer 3 and custom-synthesized at Invitrogen (their sequences are shown in Table 1). The relative levels of assayed mRNAs were calculated with comparative Ct method using GAPDH expressions as endogenous control and were normalized to sham control.

### 2.9. Evaluation of Bacterial Clearance

The bacterial loads were assessed in peritoneal cavity, liver, and lung to evaluate the bacterial clearance using a method described previously [41]. Briefly, the rats were anesthetized with ketamine and xylazine at 24 h after CLP. The peritoneal cavities were washed with 1 mL of sterile PBS and the peritoneal lavage fluids were collected under sterile condition. The livers and lungs were also removed under sterile condition. The tissues obtained were individually weighed and homogenized with sterile saline. The peritoneal fluid, liver, and lung homogenates were serially diluted with sterile PBS. 30 *μ*L of each diluted sample was placed on blood-agar base plates (Trypticase Soy Agar Deeps, Becton Dickinson, USA) and incubated at 37°C for 24 h. The numbers of bacterial colonies were then counted and expressed as colony-forming units (CFUs) per mL of peritoneal lavage or per mg of tissue.

### 2.10. Assay of Macrophage Phagocytosis

The peritoneal lavage was harvested from rats at 16 h after CLP, and peritoneal macrophages were obtained by culturing peritoneal exudate cells for 3 h and removing nonadherent cells as previously described [42]. The obtained macrophages were then plated into 24-well plates (Costar) at 1 × 10^5^ cells/well and cultured for 30 min to allow macrophages to adhere. Then the cells were incubated with carboxylate-modified fluorescent microspheres (F8827, Molecular Probe, The Netherlands) for 1 h according to manufacturer's instructions. The cells were then washed six times with cold PBS to eliminate uningested beads. Flow cytometry was performed using a FACSCalibur (BD Biosciences, San Jose, CA, USA) and the data were analyzed with the CellQuest software package (Becton Dickinson). The percentage of phagocytic cells (PP) was defined as the percentage of macrophages that ingested one or more particles. The phagocytic index (PI) was defined as the average number of particles ingested per macrophage and was calculated as follows: the total number of ingested beads was divided by the number of total macrophages.

### 2.11. Statistical Analysis

All values are expressed as mean ± SEM. All statistics analyses were performed using Prism 4.0 (GraphPad Software, USA). Differences among groups were assessed using one-way analysis of variance (ANOVA) test, followed by Dunnett's multiple comparison post hoc test. The effect of HLJDT on survival of septic rats was analyzed by Kaplan-Meier survival curves with log-rank test. *P* < 0.05 was considered statistically significant.

## 3. Results and Discussion

### 3.1. HLJDT Protects Rats from CLP-Induced Lethality and Ameliorates CLP-Induced Tissue Injuries to Livers and Lungs

**Figure 1:**
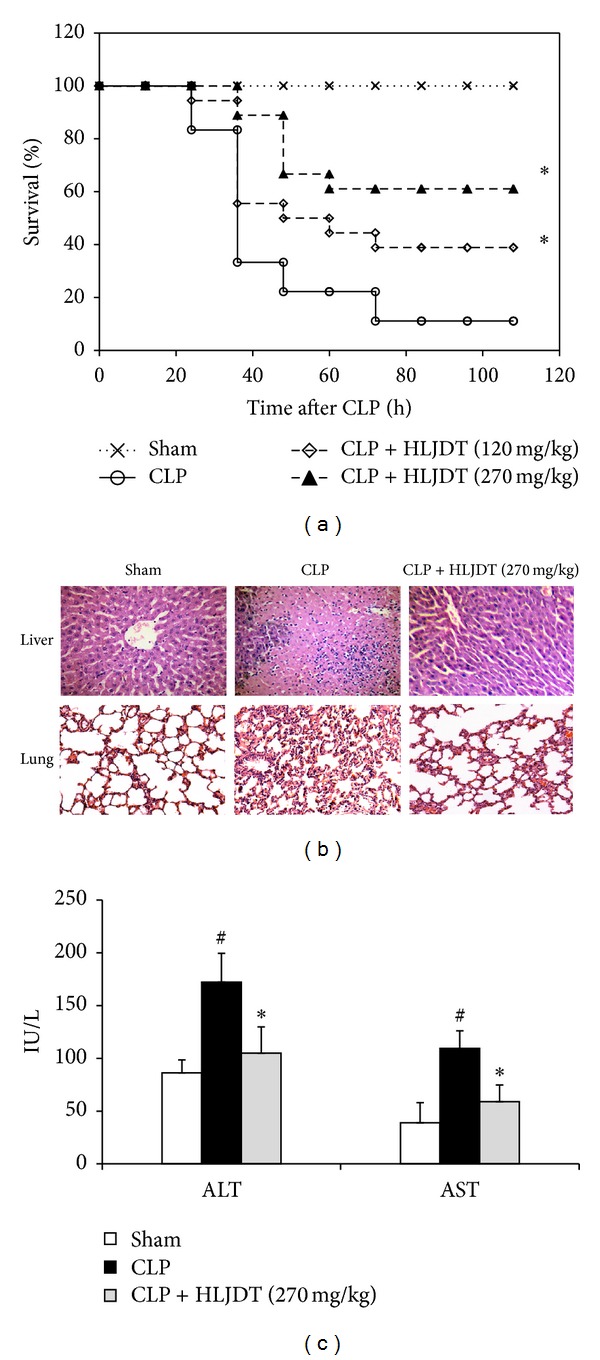
HLJDT protects rats from CLP-induced lethality and ameliorates CLP-induced tissue injuries to livers and lungs. Rats were orally administrated with HLJDT (120 or 270 mg/kg) or vehicle at 2 h prior to CLP or sham surgery. (a) Survival was monitored every 12 h for 108 h after CLP. *n* = 18 rats/group, *P < 0.05* versus* CLP control. (b) Livers and lungs were harvested 24 h after CLP for histopathologic examination using hematoxylin and eosin staining. Representative images from six animals per group were shown. (c) Blood samples were collected 24 h after CLP and ALT and AST levels in plasma were measured. *n* = 6 rats/group, *P < 0.05* versus* CLP control, ^#^ *P* < 0.05* versus *sham control.

Even though HLJDT has been shown to possess potent anti-inflammatory property [32–37], its effect on sepsis has not been investigated. In this study, an experimental sepsis was induced by CLP in rats to examine the potential therapeutic value of HLJDT for sepsis. In order to evaluate protective effect of HLJDT against sepsis, rats were treated with different doses of HLJDT (120 and 270 mg/kg) 2 h before CLP. Data of 108 h survival in HLJDT-treated and control groups are shown in Figure 1(a). Induction of sepsis by CLP resulted in 89% mortality within 108 h. The prophylactic administration of HLJDT resulted in a dose-dependent improvement in survival in septic rats (Figure 1(a)). Histopathological tests revealed that CLP induced marked histopathological changes such as inflammatory cell infiltration, congestion, necrosis, and degeneration in liver and lung of septic rats at 24 h after surgery. Administration of HLJDT (270 mg/kg) resulted in remarkable attenuation in these pathological changes (Figure 1(b)). Accordingly, CLP also caused a marked increase in the plasma levels of ALT and AST, which was significantly reduced by HLJDT treatment (Figure 1(c)). Taken together, the above results clearly demonstrated the protective effect of HLJDT against CLP-induced sepsis.

### 3.2. HLJDT Treatment Suppresses the Production of Proinflammatory Cytokines in Septic Rats

**Figure 2:**
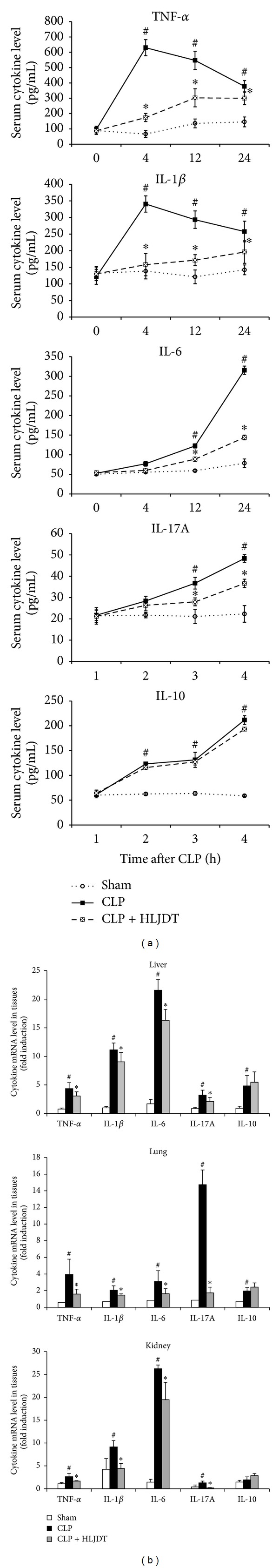
HLJDT treatment suppresses the production of proinflammatory cytokines in septic rats. Rats were orally administrated with 270 mg/kg of HLJDT or vehicle at 2 h prior to CLP or sham surgery. (a) Blood samples were collected at 0, 4, 12, and 24 h after CLP, and plasma levels of TNF-*α*, IL-1, IL-6, IL-17A, and IL-10 were measured with ELISA. (b) Liver, lung, and kidney were harvested at 24 h after CLP, and the relative mRNA levels of cytokines were analyzed by qRT-PCR. Data in (a) and (b) are mean ± SEM of 6 rats/group, *P < 0.05* versus* CLP control, ^#^ *P* < 0.05* versus* sham control.

Exaggerated host inflammatory responses are thought to contribute to tissue injury and organ dysfunction in sepsis and overproduced proinflammatory cytokines, especially TNF-*α*, IL-1, IL-6, and IL-17A, are strongly associated with sepsis syndrome. Therefore, inhibiting the overproduction of proinflammatory cytokines during early sepsis may improve sepsis outcome [3, 31, 43]. So we next investigated whether the mortality reduction by HLJDT treatment may be related to its suppression of proinflammatory cytokine production in septic rats. As shown in Figure 2(a), the plasma proinflammatory cytokines, including TNF-*α*, IL-1, IL-6, and IL-17A, were maintained in low background levels in sham control rats within 24 h after surgery. In contrast, levels of TNF-*α* and IL-1 rapidly increased after CLP surgery, peaking at 4 h, whereas the levels of IL-6 and IL-17A began to significantly increase at 12 h and continued to rise to a higher level till 24 h after CLP. The increase in TNF-*α* and IL-1 levels at 4, 12, and 24 h after CLP was markedly attenuated by HLJDT (270 mg/kg) treatment, and the levels of IL-6 and IL-17A at 12 and 24 h after CLP were also significantly suppressed by HLJDT treatment. Interestingly, even though CLP also caused a significant increase in the level of IL-10, it was not significantly affected by HLJDT treatment (Figure 2(a)). Accordingly, further determining the cytokine mRNA levels in liver, lung, and kidney by qRT-PCR revealed that CLP significantly enhanced the gene expression of these cytokines at 24 h after surgery, and treatment with 270 mg/kg of HLJDT resulted in a significant suppression of the expression of proinflammatory cytokines, including TNF-*α*, IL-1, IL-6, and IL-17A within these organs of septic rats, whereas the enhanced expression of anti-inflammatory cytokine IL-10 was not significantly affected (Figure 2(b)). These results suggested that HLJDT can selectively suppress the expression of proinflammatory cytokines, reduce the release of these inflammatory mediators into blood circulation, and therefore attenuate the systemic inflammatory responses in septic rats.

### 3.3. HLJDT Enhances Bacterial Clearance and Augments Phagocytic Activity of Macrophage in Septic Rats

**Figure 3:**
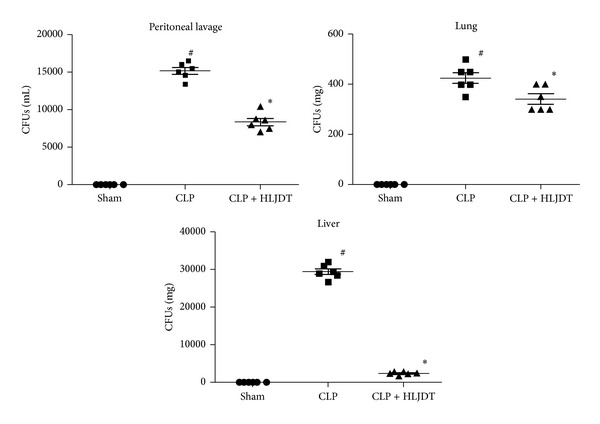
HLJDT enhances bacterial clearance in septic rats. Rats were orally administrated with 270 mg/kg of HLJDT or vehicle at 2 h prior to CLP or sham surgery. Peritoneal lavage fluid, liver, and lung samples were harvested 24 h after CLP, and the bacterial loads were determined. *n* = 6 rats/group, *P < 0.05* versus* CLP control, ^#^ *P* < 0.05* versus* sham control.

**Figure 4:**
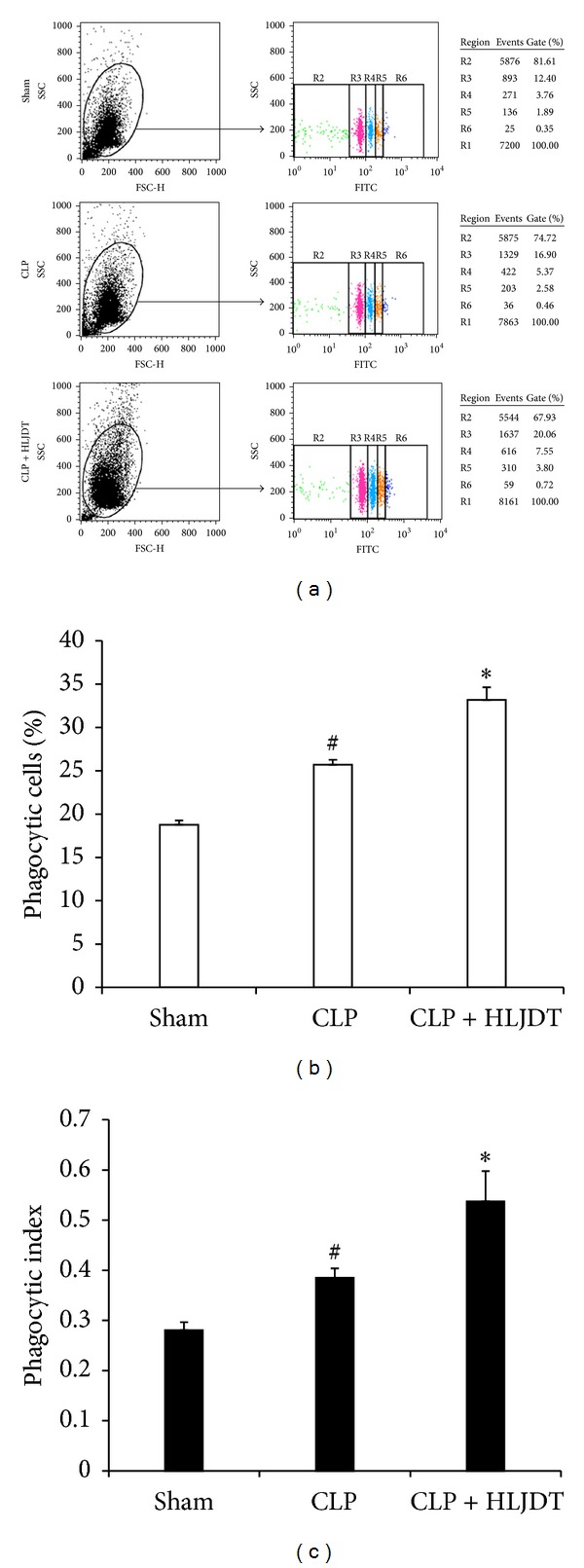
HLJDT augments phagocytic activity of macrophage in septic rats. Rats were orally administrated with 270 mg/kg of HLJDT or vehicle at 2 h prior to CLP or sham surgery. Phagocytotic activities of peritoneal macrophages were evaluated by uptake of FITC-conjugated microsphere at 16 h after CLP or sham surgery. (a) Representative results of flow cytometry analysis of phagocytosis by peritoneal macrophages after incubation with FITC-conjugated microsphere. Region 1(R1) on the dot-plot of side light scatter (SSC) *versus* forward light scatter (FSC) represented the gate of the macrophages. On the dot-plot of SSC *versus* green fluorescence (FITC), R2 represented the macrophages uningesting microspheres, and R3, R4, R5, and R6 represented the macrophages that have ingested one, two, three, and four microspheres, respectively. (b) The percentage of phagocytic cells was calculated as the percentage of macrophages that ingested one or more particles. (c) The phagocytic index was calculated as the average number of particles ingested per macrophage (the total number of ingested beads was divided by the number of total macrophages). Data in (b) and (c) are mean ± SEM of 6 rats/group, *P < 0.05* versus* CLP control, ^#^ *P* < 0.05* versus* sham control.

Although excessive production of systemic proinflammatory cytokines is believed to play a prominent role in the pathogenesis of tissue injury in sepsis, it should be acknowledged that these cytokines are integral components of host immunity against infections [44]. Therefore, the possibility exists that HLJDT treatment may lead to the impairment of host defense against infections and increase the risk of inability to eliminate the bacteria due to its supersession of cytokine production. To evaluate the effect of HLJDT on bacterial clearance in septic rats, we collected peritoneal lavage fluid, liver, and lung samples 24 h after CLP and determined the bacterial loads. The results showed that HLJDT-treated septic rats had significantly lower bacterial counts in peritoneal cavity, liver, and lung than those of untreated septic rats (Figure 3), indicating that HLJDT treatment actually enhanced bacterial clearance. Macrophages are key components of innate immunity against bacterial infections. These phagocytes mediated bacterial clearance by engulfing bacteria. We therefore further investigated whether HLJDT could affect phagocytic activity of rat macrophages using fluorescein isothiocyanate (FITC)-labeled microspheres. The results demonstrated that HLJDT significantly increased phagocytic activities of peritoneal macrophages in septic rats, as judged by percentages of fluorescent microsphere-positive cells and mean number of particles ingested per cell (phagocytic index) (Figure 4). These results suggest that the augmentation of phagocytic activities in macrophages may account, at least in part, for enhanced bacterial clearance observed in septic rats treated with HLJDT.

### 3.4. HLJDT Modulates Th1/Th2/Th17 Cytokine Profile in Spleens of Septic Rats

**Figure 5:**
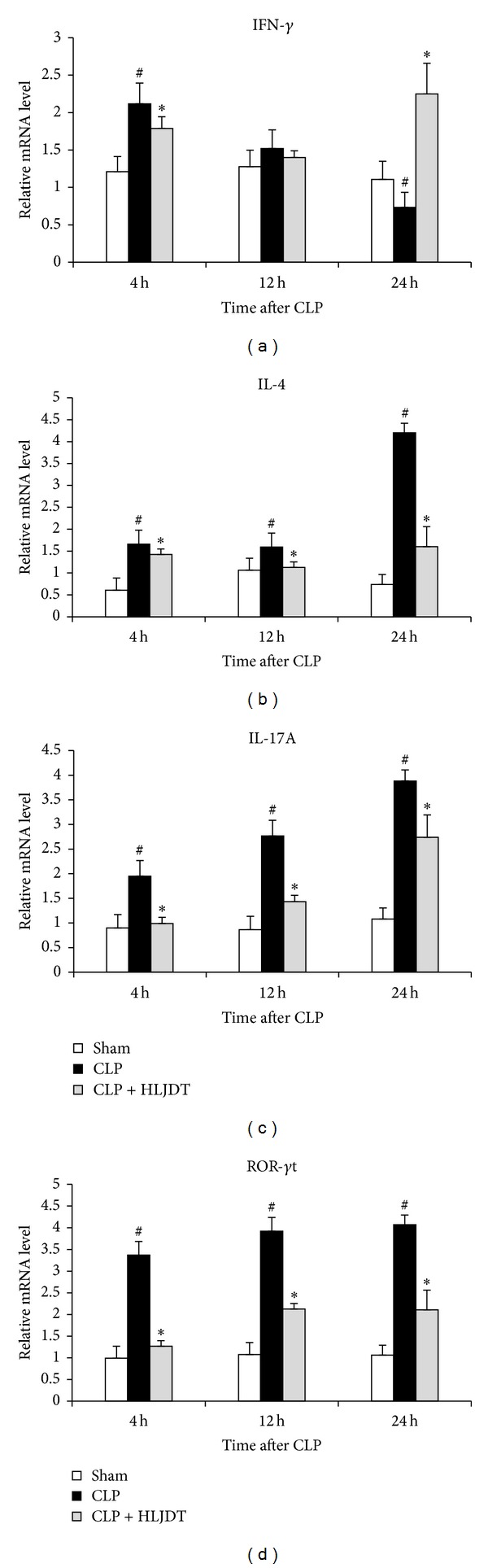
HLJDT modulates Th1/Th2/Th17 cytokine profile in spleens of septic rats. Rats were orally administrated with 270 mg/kg of HLJDT or vehicle at 2 h prior to CLP or sham surgery. The spleen samples were harvested at 4, 12, and 24 h after CLP. The relative mRNA levels of IFN-*γ* (a), IL-4 (b), IL-17A (c), and ROR-*γ*t (d) were analyzed by qRT-PCR. Data are mean ± SEM of 6 rats/group, *P < 0.05* versus* CLP control, ^#^ *P* < 0.05* versus* sham control.

T-helper (Th) cells have long been recognized as vital components of the adaptive immune system. They convey their functions by secreting a variety of cytokines and can be subdivided into different types based on their cytokine signature. Th1 cells promote cell-mediated immune responses by secreting Th1-type cytokines such as IFN-*γ* and IL-2; Th2 cells secrete cytokines such as IL-4, IL-10, and IL-5, which enhance humoral immunity [45, 46]; Th17 cells secrete IL-17 and IL-22 which have been linked to the pathogenesis of autoimmune diseases and to immune responses to bacterial and fungal infections [47, 48]. The effects of Th1 and Th2 cells are counterregulatory through their cytokine production. For example, IL-4 promotes Th2 development and inhibits Th1 cells. In contrast, IFN-*γ* promotes Th1 development and inhibits the proliferation of Th2 cells and the production of IL-4 and IL-5 by Th2 cells. It is now well established that sepsis is associated with a profound immunological dysfunction manifested by an obvious shift from Th1 to Th2 response [49–52]. Lymphocytes from the spleens of septic mice express increased levels of Th2 cytokines, which may directly or indirectly suppress the Th1 cytokine response [53, 54]. The marked depression in cell-mediated immunity results in predisposition of septic patients to secondary infections and increased mortality in sepsis [50, 51]. To investigate whether the protective effects of HLJDT in septic rats are associated with its modulation on Th1/Th2 balance in septic conditions, we examined the mRNA levels of Th1/Th2 cytokines in the spleens of HLJDT-treated and control groups. As shown in Figure 5, following CLP, the splenic IFN-*γ* mRNA level increased at first at 4 h, and declined afterwards. Till 24 h after CLP, it has dropped below the level in sham group (Figure 5(a)). Different from IFN-*γ*, the mRNA levels of IL-4 only slightly increased in spleens of septic rats within the first 12 h after CLP, but a dramatic increase was detected at 24 h after CLP. These results clearly indicated that a shift from Th1 to Th2 response was present in the septic rats following CLP (Figure 5(b)). Upon HLJDT treatment, even though the initial increase in splenic IFN-*γ* mRNA level was slightly inhibited at 4 and 12 h, the reduction of IFN-*γ* level in the later phase (at 24 h after CLP) was reversed. At the same time, the CLP-induced increase in IL-4 expression was almost completely blocked. These data indicated that HLJDT could reverse the shift from Th1 to Th2 response and promote Th1/Th2 balance toward Th1 predominance in septic rats. These data support the notion that HLJDT improved survival of septic rats as an immune-modulating agent, rather than as a simple anti-inflammatory drug. Besides Th1 and Th2 cells, Th17 cells were also found to be involved in the pathogenesis of sepsis. It was demonstrated that the CLP induced increased IL-17A production in mice and neutralization of IL-17A correlated with decreased bacteremia, increased survival, and decreased plasma levels of proinflammatory cytokines TNF-*α*, IL-1*β*, and IL-6 [9]. In another study, CLP also increased levels of IL-22, the other hallmark cytokine of Th17 cells, in spleen and kidney of septic mice and inhibition of IL-22 following CLP enhanced bacterial clearance, promoted phagocyte recruitment, and attenuated organ dysfunction [55]. In addition, clinical data from patients with abdominal sepsis suggest that higher levels of IL-22 may facilitate bacterial burden and septic complications [56]. Together, these data suggest that perturbation of Th17 activation in septic conditions negatively impacts outcomes from sepsis. In the present study, we have revealed that CLP induced a significant increase in plasma IL-17A level as well as IL-17A mRNA level in target organs including livers, lungs, and kidneys of septic rats, which were all inhibited by HLJDT treatment. These results suggest that CLP-induced Th17 activation is suppressed by HLJDT. To provide more evidence, we further detected IL-17A mRNA levels in spleens of HLJDT-treated and control groups and found that the IL-17A mRNA level progressively increased in spleens of septic rats from 4 h to 24 h following CLP, which was significantly suppressed by HLJDT treatment (Figure 5(c)). Since the transcription factor ROR-*γ*t is a “master gene” in controlling IL-17 and IL-22 expression in Th17 cells [48], we also examined ROR-*γ*t mRNA level in the spleens. Accordingly, the splenic ROR-*γ*t mRNA level was markedly elevated following CLP and was significantly suppressed upon HLJDT treatment (Figure 5(d)). Taken together, our data indicated HLJDT is able to inhibit the activation of Th17 cells in septic condition. Since overactivated Th17 has been proposed to have adverse functions in sepsis, it is reasonable to speculate that the protective effects of HLJDT against sepsis partially come from its inhibition of Th17.

## 4. Conclusion

In conclusion, our results showed that prophylactic administration of HLJDT protects rats from CLP-induced lethality and ameliorates CLP-induced liver and lung injury. The protective effect of HLJDT against sepsis seems to be associated with its intervention on several different aspects of sepsis, including attenuating excessive inflammatory response through suppression of the production of proinflammatory cytokines, facilitating bacterial clearance by augmentation of phagocytic activity of macrophage, preserving adaptive immune reaction by reversal of sepsis-induced shift from Th1 to Th2 response, and inhibiting Th17 activation in septic conditions. Thus, we propose that HLJDT is a potential medication for septic patients.

## Figures and Tables

**Table 1:** The sequences of primers used in this study.

Gene name	Forward primer sequences	Reverse primer sequences
GAPDH	CGCATCTTCTTGTGCAGTGCCAGCC	TTGTCACAAGAGAAGGCAGCCCTGG
TNF-*α*	ACTGAACTTCGGGGTGATCGGT	TGGTTTGCTACGACGTGGGCTA
IL-1*β*	AATGCCTCGTGCTGTCTGACCCAT	CCAAGGCCACAGGGATTTTGTCGTT
IL-6	ACCACTTCACAAGTCGGAGGCTT	CTGACAGTGCATCATCGCTGTTCA
IL-10	AAAAGCAAGGCAGTGGAGCAGGTG	TGGCCTTGTAGACACCTTTGTCTTG
IL-17A	ACCTCAACCGTTCCACTTCACCCT	ATGTGGTGGTCCAACTTCCCCTCA
IFN-*γ*	GACAACCAGGCCATCAGCAACAACA	CAGCTTTGTGCTGGATCTGTGGGT
IL-4	GAACAAGTCTGGGGTTCTCG	TTGTGAGCGTGGACTCATTC
ROR-*γ*t	TCTGGAAGCTGTGGGATAGA	GAGGAGCCTGTGGAGAAATAC
